# Nonapiperidinium monohydrogen deca­vanadate tetra­nitrate

**DOI:** 10.1107/S1600536809026555

**Published:** 2009-07-18

**Authors:** Mohsen Graia, Regaya Ksiksi, Ahmed Driss

**Affiliations:** a Laboratoire de Matériaux et de Cristallochimie, Faculté des Sciences de Tunis, Université de Tunis–El Manar, 2092 El Manar II Tunis, Tunisia

## Abstract

The title compound, (C_5_H_12_N)_9_[HV_10_O_28_](NO_3_)_4_, contains a monoprotonated deca­vanadate polyanion which lies on an inversion center. All the piperidinium cations adopt chair conformations. In the crystal structure, inter­molecular N—H⋯O hydrogen bonds form chains along [001]. As well as half of a polyanion, the asymmetric unit contains one full and two half-occupancy nitrate ions and four full occupancy and one half-occupancy piperidinium cations; the half-occupancy piperidinium cation is disordered over two general sites with occupancies of 0.32 and 0.18, and is, in turn, disordered over an inversion center.

## Related literature

For the biological activity of vanadium, see: Crans (1994 ▶); Elvingson *et al.* (1996 ▶). For its inter­actions with nitro­gen compounds such as proteins and amino acids and its role in enzymatic reactions, see: Correia *et al.* (2004 ▶). For related structures, see: Ferreira da Silva *et al.* (2003 ▶); Maciejewska *et al.* (2003 ▶); Arrieta (1992 ▶); Wang *et al.* (2008 ▶); Wery *et al.* (1996 ▶). 
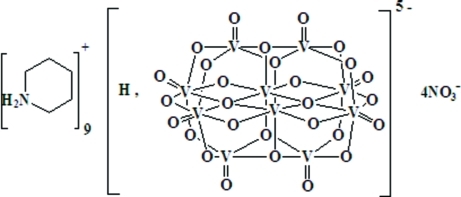


## Experimental

### 

#### Crystal data

(C_5_H_12_N)_9_[HV_10_O_28_](NO_3_)_4_
*M* *_r_* = 1981.85
Triclinic, 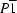

*a* = 11.593 (2) Å
*b* = 13.290 (2) Å
*c* = 14.676 (2) Å
α = 105.858 (2)°
β = 110.335 (2)°
γ = 92.457 (2)°
*V* = 2015.6 (5) Å^3^
*Z* = 1
Mo *K*α radiation
μ = 1.20 mm^−1^
*T* = 293 K
0.30 × 0.25 × 0.14 mm

#### Data collection

Enraf–Nonius CAD-4 diffractometer
Absorption correction: ψ scan (North *et al*., 1968 ▶) *T*_min_ = 0.72, *T*_max_ = 0.90 (expected range = 0.676–0.846)
9193 measured reflections
8755 independent reflections
6099 reflections with *I* > 2σ(*I*)
*R*_int_ = 0.047
2 standard reflections frequency: 120 min intensity decay: 2%

#### Refinement

*R*[*F* ^2^ > 2σ(*F* ^2^)] = 0.043
*wR*(*F* ^2^) = 0.118
*S* = 1.04
8755 reflections
598 parameters
262 restraints
H-atom parameters constrained
Δρ_max_ = 0.71 e Å^−3^
Δρ_min_ = −0.32 e Å^−3^

### 

Data collection: *CAD-4 EXPRESS* (Duisenberg, 1992 ▶; Macíček & Yordanov, 1992 ▶); cell refinement: *CAD-4 EXPRESS*; data reduction: *MolEN* (Fair, 1990 ▶); program(s) used to solve structure: *SHELXS97* (Sheldrick, 2008 ▶); program(s) used to refine structure: *SHELXL97* (Sheldrick, 2008 ▶); molecular graphics: *DIAMOND* (Brandenburg, 1998 ▶); software used to prepare material for publication: *publCIF* (Westrip, 2009 ▶).

## Supplementary Material

Crystal structure: contains datablocks I, global. DOI: 10.1107/S1600536809026555/lh2828sup1.cif

Structure factors: contains datablocks I. DOI: 10.1107/S1600536809026555/lh2828Isup2.hkl

Additional supplementary materials:  crystallographic information; 3D view; checkCIF report

## Figures and Tables

**Table 1:** Hydrogen-bond geometry (Å, °)

*D*—H⋯*A*	*D*—H	H⋯*A*	*D*⋯*A*	*D*—H⋯*A*
N*C*1—H*C*1*A*⋯O8	0.90	1.80	2.693 (3)	174
N*C*1—H*C*1*B*⋯O*N*6*A*^i^	0.90	1.95	2.829 (8)	164
N*C*1—H*C*1*B*⋯O*N*6*B*	0.90	2.05	2.876 (9)	153
N*C*1—H*C*1*B*⋯O*N*5*A*^i^	0.90	2.48	3.214 (12)	139
N*C*1—H*C*1*B*⋯O*N*5*B*	0.90	2.54	3.355 (13)	151
N*C*2—H*C*2*A*⋯O*N*4*A*	0.90	1.95	2.824 (7)	164
N*C*2—H*C*2*A*⋯O*N*4*B*^i^	0.90	2.05	2.909 (8)	159
N*C*2—H*C*2*A*⋯O*N*5*B*^i^	0.90	2.50	3.280 (13)	145
N*C*2—H*C*2*A*⋯O*N*5*A*	0.90	2.56	3.251 (12)	134
N*C*2—H*C*2*B*⋯O4	0.90	1.85	2.746 (3)	174
N*C*3—H*C*3*A*⋯O5^ii^	0.90	1.85	2.749 (4)	175
N*C*3—H*C*3*B*⋯O*N*3	0.90	2.06	2.885 (5)	152
N*C*3—H*C*3*B*⋯O*N*2	0.90	2.31	3.095 (5)	145
N*C*4—H*C*4*A*⋯O7	0.90	1.82	2.716 (4)	172
N*C*4—H*C*4*B*⋯O*N*3	0.90	2.16	2.954 (6)	147
N*C*4—H*C*4*B*⋯O*N*1	0.90	2.26	3.060 (6)	148
N*C*5—H*C*5*A*⋯O6	0.90	2.40	3.248 (17)	158
N*C*6—H*C*6*A*⋯O*N*4*B*	0.90	2.12	2.92 (2)	147
N*C*6—H*C*6*B*⋯O6^i^	0.90	1.92	2.80 (2)	166

Symmetry codes: (i) 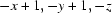; (ii) 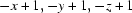.

**Fig. 1.**
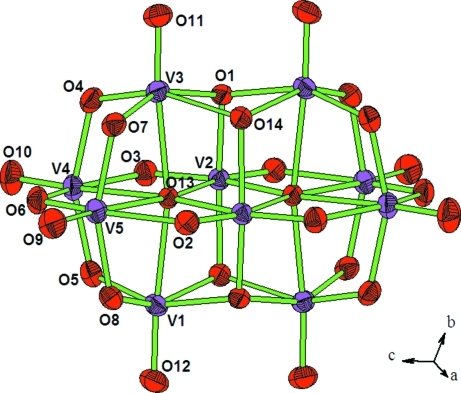
View of the decavanadate unit of the title compound. Thermal ellipsoids are drawn at 35% probability. The purple spheres are vanadium atoms and the red spheres are oxygen atoms.

**Fig. 2.**
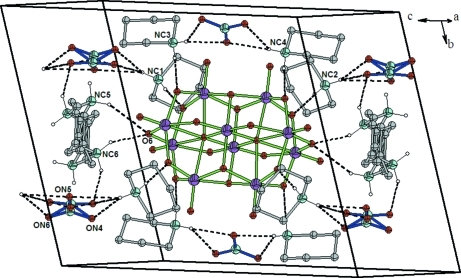
Part of the crystal structure of the title compound. The purple spheres are vanadium atoms, the red spheres are oxygen atoms, the cyan spheres are nitrogen atoms, the green spheres are carbon atoms and the white spheres represent hydrogen atoms. Hydrogen bonds are shown as dashed lines.

## supplementary crystallographic information

### Comment

Vanadium is a rare metal with exceptional properties. Both its cationic and anionic forms can interact with biomolecules, and its coordination chemistry plays a predominant role in these interactions. Among several biological functions of vanadium, many important therapeutic effects have been described, including hormonal, cardiovascular, anticarcinogenic, sugar lowering activities (Elvingson *et al.*,  1996; Crans, 1994). Because of the physiological relevance of vanadium, a better understanding of its complexation behavior with organic ligands is of vital interest. The interactions of this metal with nitrogen compounds like proteins and amino acids and its role in enzymatic reactions have been studied extensively (Correia *et al.*,  2004). Herein we present the crystal structure of the title compound (I).

The asymmetric unit of (I) contains one half of a monoprotonated decavanadate polyanion [HV_10_O_28_]^5-^, 4.5 piperidinum cations (C_5_H_12_N^+^), and 2 NO_3_^-^anions. The formula unit is generated by a  crystallographic inversion centre. The [HV_10_O_28_]^5-^ polyanion is composed of ten distorted VO_6_ edge-sharing octahedra and is best described as cubic close-packing of oxygen ions, with the octahedral holes filled by vanadium ions. Each VO_6_ octahedron is considerably distorted, with bond angles at the V atoms ranging from 1.602 (2) to 2.345 (2) Å. The V—O distance depends upon the type of oxo ligands: V=O_t_ bond lengths to the terminal oxo O atoms vary from 1.603 (3) to 1.608 (2) Å, V—O_2b_ bond lengths to the O atoms bridging two V atoms vary from 1.693 (3) to 2.059 (3) Å, V—O_3b_ bond lengths to the O atoms bridging three V atoms vary from 1.914 (2) to 2.067 (3) Å and V—O_6b_ bond lengths to the O atoms shared between six V atoms range 2.081 (3) to 2.345 (3) Å. The V—V distances are in the range 3.091 (4) to 3.286 (4) Å. The V—O bond and angles of the [HV_10_O_28_]^6-^ are in agreement with those reported in literature (Ferreira da Silva *et al.*, 2003; Maciejewska *et al.*,  2003; Arrieta, 1992).

The organic groups are present as cations, C_5_H_12_N^+^. These piperidinium rings adopt chair conformation (Fig. 2). The bond lengths of C–N and C–C are in the range of 1.468 (6) – 1.502 (7) Å and 1.469 (8) – 1.543 (7) Å, respectively. The C–C–C, C—C—N and C—N—C angles are in the range of 106 (1) – 113 (1) Å, 107 (2) – 111.7 (4) Å and 112.1 (4) – 114.1 (1) Å, respectively. These values are in agreement with those reported in literature (Wang *et al.*,  2008). As a result, we found one of the piperidinium cations in special position; this cation is disordred with a *ca* 16:9 occupancy ratio for its (NC5, C_5_H_12_N^+^) and (NC6, C_5_H_12_N^+^) components.

Similarly, we identified one disordered nitrate group, with a similar occupancy ratio for components N2O_3_A and N2O_3_B. The central N atom of N1O_3_, N2O_3_A and of N2O_3_B nitrate groups is close to coplanarity with the three attached O atoms. The largest deviation from the plane being 0.0004 Å, 0.0062 and 0.0064 respectively. The N–O bond distances and O–N–O angles are in agreement with in the nitrate unit.

The most important feature of this crystal is the presence of N–H···O, hydrogen bonds with D···A distances ranging from 2.693 (3) to 3.355 (13) Å. These interactions connect the various fragments into a supramolecular structure. In fact, it is noted that piperidinium C_5_H_12_N^+ ^cations are located around the [HV_10_O_28_] (Fig. 2). Each [HV_10_O_28_]^5-^ cluster is surrounded by ten C_5_H_12_N^+^ cations. The N atoms of the organic cations are directing towards the doubly bridging O atoms of the cluster anion there by forming strong H-bonding. The NO_3_^-^anions contribute to the cohesion of the structure by hydrogen bonds (Fig 2). In fact, as can be seen from the packing diagram (Fig. 2), there are intermolecular hydrogen bonds between the nitrato O atoms and the N–H group of the piperidinium C_5_H_12_N^+^ cations.

### Experimental

The title compound was prepared by the reaction of vanadium (V) oxide (0.68 g, 3.74 mmol, Fluka, 99,9%), piperidin (1.72 g, 20.23 mmol, Fluka, > 99%), zinc nitrate (1.12 g, 3.77 mmol,Fluka, > 99%) and oxalic acid (1.23 g, 9.77 mmol, Prolabo, > 98%), dissolved in 40 ml ofdistilled water. Orange single crystals were obtained after six days from by slow evaporation at room temperature.

### Refinement

The positions of the H atoms attached to the piperidinium cations were placed at geometrically idealized positions (C–H = 0.97 Å, N–H =0.90 Å) and constrained to ride on their parent atoms with *U*_iso_(H)= 1.2*U*_eq_(carrier atom). The hydrogen atom attached to the [V_10_O_28_] cluster could not be located but is included in the molecular formula. The disordered model was refined by using the tools available in the *SHELXL97* (Sheldrick, 2008) software.

### Crystal data

**Table:**

(C_5_H_12_N)_9_[HV_10_O_28_](NO_3_)_4_	*Z* = 1
*M**_r_* = 1981.85	*F*(000) = 1020
Triclinic, *P*1	*D*_x_ = 1.633 Mg m^−^^3^
Hall symbol: -P 1	Mo *K*α radiation, λ = 0.71073 Å
*a* = 11.593 (2) Å	Cell parameters from 25 reflections
*b* = 13.290 (2) Å	θ = 11.8–15.2°
*c* = 14.676 (2) Å	µ = 1.20 mm^−^^1^
α = 105.858 (2)°	*T* = 293 K
β = 110.335 (2)°	Hexagone, orange
γ = 92.457 (2)°	0.30 × 0.25 × 0.14 mm
*V* = 2015.6 (5) Å^3^	

### Data collection

**Table:**

Enraf–Nonius CAD-4 diffractometer	6099 reflections with *I* > 2σ(*I*)
Radiation source: fine-focus sealed tube	*R*_int_ = 0.047
graphite	θ_max_ = 27.0°, θ_min_ = 2.3°
ω/2θ scans	*h* = 0→14
Absorption correction: ψ scan (North et al., 1968)	*k* = −16→16
*T*_min_ = 0.72, *T*_max_ = 0.90	*l* = −18→17
9193 measured reflections	2 standard reflections every 120 min
8755 independent reflections	intensity decay: 2%

### Refinement

**Table:**

Refinement on *F*^2^	Primary atom site location: structure-invariant direct methods
Least-squares matrix: full	Secondary atom site location: difference Fourier map
*R*[*F*^2^ > 2σ(*F*^2^)] = 0.043	Hydrogen site location: inferred from neighbouring sites
*wR*(*F*^2^) = 0.118	H-atom parameters constrained
*S* = 1.04	*w* = 1/[σ^2^(*F*_o_^2^) + (0.0517*P*)^2^ + 1.3683*P*] where *P* = (*F*_o_^2^ + 2*F*_c_^2^)/3
8755 reflections	(Δ/σ)_max_ = 0.001
598 parameters	Δρ_max_ = 0.71 e Å^−^^3^
262 restraints	Δρ_min_ = −0.32 e Å^−^^3^

### Special details

**Table:**

Geometry. All e.s.d.'s (except the e.s.d. in the dihedral angle between two l.s. planes) are estimated using the full covariance matrix. The cell e.s.d.'s are taken into account individually in the estimation of e.s.d.'s in distances, angles and torsion angles; correlations between e.s.d.'s in cell parameters are only used when they are defined by crystal symmetry. An approximate (isotropic) treatment of cell e.s.d.'s is used for estimating e.s.d.'s involving l.s. planes.
Refinement. Refinement of *F*^2^ against ALL reflections. The weighted *R*-factor *wR* and goodness of fit *S* are based on *F*^2^, conventional *R*-factors *R* are based on *F*, with *F* set to zero for negative *F*^2^. The threshold expression of *F*^2^ > σ(*F*^2^) is used only for calculating *R*-factors(gt) *etc*. and is not relevant to the choice of reflections for refinement. *R*-factors based on *F*^2^ are statistically about twice as large as those based on *F*, and *R*- factors based on ALL data will be even larger.

### Fractional atomic coordinates and isotropic or equivalent isotropic displacement parameters (Å^2^)

**Table:**

	*x*	*y*	*z*	*U*_iso_*/*U*_eq_	Occ. (<1)
V1	0.55004 (5)	0.32924 (4)	0.57070 (4)	0.03398 (13)	
V2	0.65119 (4)	0.53839 (4)	0.54702 (4)	0.03033 (12)	
V3	0.53337 (5)	0.33546 (4)	0.35568 (4)	0.03371 (13)	
V4	0.62554 (5)	0.54954 (4)	0.33350 (4)	0.03911 (14)	
V5	0.34440 (5)	0.46252 (4)	0.24253 (4)	0.03735 (14)	
O1	0.64328 (19)	0.38796 (15)	0.50197 (15)	0.0347 (5)	
O2	0.75038 (19)	0.56505 (17)	0.66911 (15)	0.0381 (5)	
O3	0.73307 (19)	0.56874 (17)	0.48006 (16)	0.0398 (5)	
O4	0.63901 (19)	0.40224 (17)	0.31868 (16)	0.0402 (5)	
O5	0.5792 (2)	0.67820 (17)	0.38378 (16)	0.0405 (5)	
O6	0.4801 (2)	0.50774 (18)	0.21948 (15)	0.0442 (5)	
O7	0.39219 (19)	0.33201 (16)	0.24371 (15)	0.0384 (5)	
O8	0.33299 (19)	0.60716 (17)	0.30494 (15)	0.0388 (5)	
O9	0.2324 (2)	0.4357 (2)	0.13345 (16)	0.0544 (6)	
O10	0.7271 (2)	0.5810 (2)	0.29078 (19)	0.0591 (7)	
O11	0.5568 (2)	0.21462 (17)	0.32664 (17)	0.0479 (6)	
O12	0.5744 (2)	0.20838 (17)	0.54415 (18)	0.0493 (6)	
O13	0.50819 (17)	0.49809 (15)	0.59153 (14)	0.0329 (4)	
O14	0.41649 (19)	0.32453 (16)	0.43204 (15)	0.0377 (5)	
NO1	0.0877 (6)	0.0576 (4)	0.2869 (3)	0.0928 (15)	
ON1	0.0314 (4)	0.0356 (4)	0.1946 (3)	0.1313 (17)	
ON2	0.0455 (4)	0.0246 (3)	0.3412 (3)	0.1027 (12)	
ON3	0.1908 (5)	0.1153 (3)	0.3274 (3)	0.1214 (17)	
NO2A	0.7294 (9)	0.2038 (8)	−0.0230 (6)	0.067 (4)	0.50
ON4A	0.6735 (8)	0.1750 (5)	0.0262 (5)	0.081 (2)	0.50
ON5A	0.8303 (11)	0.2627 (12)	0.0230 (8)	0.118 (6)	0.50
ON6A	0.6819 (10)	0.1762 (6)	−0.1164 (5)	0.095 (3)	0.50
NO2B	0.3155 (9)	0.7704 (8)	0.0289 (7)	0.069 (3)	0.50
ON4B	0.3787 (7)	0.7673 (7)	−0.0239 (5)	0.088 (2)	0.50
ON5B	0.2040 (8)	0.7800 (15)	−0.0057 (10)	0.111 (5)	0.50
ON6B	0.3636 (8)	0.7675 (8)	0.1167 (5)	0.097 (3)	0.50
NC1	0.1785 (3)	0.7091 (2)	0.1898 (2)	0.0501 (7)	
HC1A	0.2326	0.6740	0.2251	0.060*	
HC1B	0.2150	0.7357	0.1546	0.060*	
C11	0.1520 (4)	0.7977 (3)	0.2628 (3)	0.0617 (10)	
H11A	0.0981	0.8390	0.2259	0.074*	
H11B	0.2291	0.8438	0.3095	0.074*	
C12	0.0900 (4)	0.7552 (4)	0.3221 (4)	0.0780 (13)	
H12A	0.0693	0.8134	0.3672	0.094*	
H12B	0.1472	0.7196	0.3639	0.094*	
C13	−0.0280 (4)	0.6778 (4)	0.2506 (5)	0.0964 (17)	
H13A	−0.0632	0.6474	0.2899	0.116*	
H13B	−0.0890	0.7150	0.2142	0.116*	
C14	0.0021 (4)	0.5903 (4)	0.1746 (4)	0.0895 (16)	
H14A	0.0558	0.5485	0.2109	0.107*	
H14B	−0.0743	0.5440	0.1268	0.107*	
C15	0.0651 (4)	0.6333 (3)	0.1166 (3)	0.0719 (12)	
H15A	0.0878	0.5758	0.0723	0.086*	
H15B	0.0087	0.6690	0.0743	0.086*	
NC2	0.7900 (3)	0.2878 (2)	0.2361 (2)	0.0481 (7)	
HC2A	0.7548	0.2636	0.1675	0.058*	
HC2B	0.7356	0.3223	0.2592	0.058*	
C21	0.8144 (4)	0.1962 (3)	0.2766 (3)	0.0592 (10)	
H21A	0.8686	0.1558	0.2485	0.071*	
H21B	0.7367	0.1501	0.2561	0.071*	
C22	0.8749 (4)	0.2352 (3)	0.3914 (3)	0.0674 (11)	
H22A	0.8949	0.1753	0.4166	0.081*	
H22B	0.8167	0.2687	0.4193	0.081*	
C23	0.9934 (4)	0.3139 (3)	0.4281 (4)	0.0705 (12)	
H23A	1.0562	0.2785	0.4080	0.085*	
H23B	1.0257	0.3423	0.5020	0.085*	
C24	0.9644 (4)	0.4038 (3)	0.3813 (3)	0.0666 (11)	
H24A	0.9089	0.4438	0.4079	0.080*	
H24B	1.0409	0.4512	0.4010	0.080*	
C25	0.9051 (4)	0.3631 (3)	0.2668 (3)	0.0665 (11)	
H25A	0.8849	0.4217	0.2396	0.080*	
H25B	0.9626	0.3277	0.2392	0.080*	
NC3	0.2756 (3)	0.1265 (2)	0.5400 (2)	0.0582 (8)	
HC3A	0.3266	0.1885	0.5636	0.070*	
HC3B	0.2239	0.1196	0.4758	0.070*	
C31	0.3514 (4)	0.0395 (3)	0.5372 (3)	0.0701 (12)	
H31A	0.3995	0.0433	0.4955	0.084*	
H31B	0.2971	−0.0282	0.5069	0.084*	
C32	0.4371 (4)	0.0478 (4)	0.6429 (4)	0.0754 (13)	
H32A	0.4980	0.1113	0.6696	0.091*	
H32B	0.4814	−0.0126	0.6407	0.091*	
C33	0.3668 (5)	0.0516 (4)	0.7133 (4)	0.0879 (15)	
H33A	0.4257	0.0642	0.7825	0.105*	
H33B	0.3151	−0.0162	0.6927	0.105*	
C34	0.2857 (5)	0.1383 (4)	0.7110 (4)	0.0842 (14)	
H34A	0.2358	0.1340	0.7512	0.101*	
H34B	0.3385	0.2067	0.7418	0.101*	
C35	0.2012 (4)	0.1296 (4)	0.6038 (4)	0.0799 (14)	
H35A	0.1405	0.0658	0.5758	0.096*	
H35B	0.1568	0.1898	0.6045	0.096*	
NC4	0.2310 (4)	0.1475 (3)	0.1487 (3)	0.0796 (11)	
HC4A	0.2795	0.2109	0.1827	0.096*	
HC4B	0.1871	0.1355	0.1856	0.096*	
C41	0.1427 (5)	0.1505 (4)	0.0468 (5)	0.111 (2)	
H41A	0.0856	0.0843	0.0122	0.133*	
H41B	0.0943	0.2073	0.0568	0.133*	
C42	0.2114 (6)	0.1669 (5)	−0.0166 (5)	0.114 (2)	
H42A	0.2619	0.2364	0.0150	0.137*	
H42B	0.1526	0.1651	−0.0830	0.137*	
C43	0.2948 (6)	0.0835 (5)	−0.0303 (4)	0.119 (2)	
H43A	0.3442	0.1007	−0.0669	0.143*	
H43B	0.2441	0.0150	−0.0701	0.143*	
C44	0.3815 (5)	0.0786 (5)	0.0750 (4)	0.0982 (17)	
H44A	0.4276	0.0200	0.0655	0.118*	
H44B	0.4409	0.1434	0.1102	0.118*	
C45	0.3101 (5)	0.0651 (4)	0.1384 (4)	0.0896 (16)	
H45A	0.2585	−0.0038	0.1075	0.108*	
H45B	0.3676	0.0678	0.2056	0.108*	
NC5	0.4959	0.6247 (10)	0.0557 (12)	0.074 (4)	0.32
HC5A	0.4993	0.6110	0.1133	0.089*	0.32
HC5B	0.4890	0.6935	0.0644	0.089*	0.32
C51	0.3848	0.5597 (14)	−0.0301 (17)	0.070 (6)	0.32
H51A	0.3801	0.5738	−0.0926	0.084*	0.32
H51B	0.3104	0.5774	−0.0173	0.084*	0.32
C52	0.3927	0.4430 (15)	−0.0415 (18)	0.054 (5)	0.32
H52A	0.3941	0.4273	0.0196	0.065*	0.32
H52B	0.3219	0.3988	−0.0993	0.065*	0.32
C53	0.5118	0.4239 (9)	−0.0582 (11)	0.051 (3)	0.32
H53A	0.5073	0.4388	−0.1203	0.061*	0.32
H53B	0.5198	0.3497	−0.0678	0.061*	0.32
C54	0.6266	0.4906 (16)	0.0293 (16)	0.063 (5)	0.32
H54A	0.6338	0.4759	0.0920	0.075*	0.32
H54B	0.7008	0.4749	0.0151	0.075*	0.32
C55	0.6128	0.6047 (15)	0.0395 (16)	0.068 (5)	0.32
H55A	0.6830	0.6503	0.0968	0.081*	0.32
H55B	0.6108	0.6200	−0.0219	0.081*	0.32
NC6	0.493 (2)	0.5751 (14)	−0.0322 (17)	0.061 (5)	0.18
HC6A	0.4901	0.6450	−0.0147	0.073*	0.18
HC6B	0.4911	0.5541	−0.0964	0.073*	0.18
C61	0.383 (2)	0.519 (2)	−0.029 (3)	0.067 (8)	0.18
H61A	0.3071	0.5352	−0.0734	0.080*	0.18
H61B	0.3835	0.5401	0.0403	0.080*	0.18
C62	0.389 (3)	0.402 (2)	−0.064 (2)	0.055 (7)	0.18
H62A	0.3159	0.3613	−0.0652	0.066*	0.18
H62B	0.3897	0.3813	−0.1321	0.066*	0.18
C63	0.505 (3)	0.3777 (17)	0.0089 (19)	0.061 (5)	0.18
H63A	0.5076	0.3023	−0.0104	0.073*	0.18
H63B	0.5062	0.4006	0.0779	0.073*	0.18
C64	0.617 (3)	0.438 (2)	0.003 (3)	0.072 (8)	0.18
H64A	0.6942	0.4223	0.0473	0.087*	0.18
H64B	0.6150	0.4160	−0.0661	0.087*	0.18
C65	0.611 (2)	0.555 (2)	0.038 (3)	0.058 (9)	0.18
H65A	0.6132	0.5762	0.1071	0.070*	0.18
H65B	0.6811	0.5952	0.0359	0.070*	0.18

### Atomic displacement parameters (Å^2^)

**Table:**

	*U*^11^	*U*^22^	*U*^33^	*U*^12^	*U*^13^	*U*^23^
V1	0.0360 (3)	0.0331 (3)	0.0323 (3)	0.0080 (2)	0.0090 (2)	0.0139 (2)
V2	0.0313 (3)	0.0305 (3)	0.0285 (2)	0.0043 (2)	0.0100 (2)	0.0095 (2)
V3	0.0336 (3)	0.0355 (3)	0.0308 (3)	0.0096 (2)	0.0113 (2)	0.0085 (2)
V4	0.0404 (3)	0.0491 (3)	0.0362 (3)	0.0076 (2)	0.0195 (2)	0.0192 (2)
V5	0.0390 (3)	0.0440 (3)	0.0252 (2)	0.0100 (2)	0.0059 (2)	0.0120 (2)
O1	0.0392 (11)	0.0362 (11)	0.0318 (10)	0.0157 (9)	0.0136 (9)	0.0136 (9)
O2	0.0321 (11)	0.0453 (12)	0.0323 (11)	0.0086 (9)	0.0066 (9)	0.0113 (9)
O3	0.0334 (11)	0.0506 (13)	0.0406 (12)	0.0092 (10)	0.0163 (9)	0.0183 (10)
O4	0.0382 (12)	0.0498 (13)	0.0379 (11)	0.0129 (10)	0.0202 (10)	0.0131 (10)
O5	0.0437 (12)	0.0438 (12)	0.0401 (12)	0.0048 (10)	0.0172 (10)	0.0206 (10)
O6	0.0491 (13)	0.0581 (14)	0.0289 (11)	0.0119 (11)	0.0156 (10)	0.0171 (10)
O7	0.0400 (12)	0.0407 (12)	0.0281 (10)	0.0099 (9)	0.0086 (9)	0.0057 (9)
O8	0.0384 (12)	0.0450 (12)	0.0325 (11)	0.0125 (9)	0.0073 (9)	0.0180 (9)
O9	0.0534 (15)	0.0671 (16)	0.0293 (11)	0.0154 (12)	0.0007 (10)	0.0125 (11)
O10	0.0555 (15)	0.0805 (18)	0.0568 (15)	0.0059 (13)	0.0327 (13)	0.0307 (14)
O11	0.0520 (14)	0.0419 (13)	0.0459 (13)	0.0170 (11)	0.0162 (11)	0.0085 (10)
O12	0.0586 (15)	0.0373 (12)	0.0521 (14)	0.0147 (11)	0.0158 (12)	0.0196 (11)
O13	0.0334 (11)	0.0424 (11)	0.0284 (10)	0.0150 (9)	0.0135 (9)	0.0157 (9)
O14	0.0408 (12)	0.0439 (12)	0.0348 (11)	0.0174 (10)	0.0160 (9)	0.0180 (9)
NO1	0.134 (4)	0.092 (3)	0.064 (3)	0.068 (3)	0.041 (3)	0.030 (3)
ON1	0.117 (3)	0.198 (5)	0.060 (2)	0.044 (3)	0.014 (2)	0.033 (3)
ON2	0.116 (3)	0.110 (3)	0.095 (3)	0.043 (2)	0.049 (3)	0.035 (2)
ON3	0.186 (5)	0.098 (3)	0.058 (2)	−0.006 (3)	0.024 (3)	0.022 (2)
NO2A	0.110 (12)	0.045 (6)	0.053 (6)	0.020 (7)	0.033 (8)	0.019 (4)
ON4A	0.142 (7)	0.053 (4)	0.055 (4)	0.003 (4)	0.048 (4)	0.013 (3)
ON5A	0.111 (8)	0.157 (15)	0.083 (7)	−0.008 (7)	0.033 (6)	0.039 (8)
ON6A	0.178 (10)	0.057 (4)	0.051 (4)	0.002 (5)	0.045 (5)	0.017 (3)
NO2B	0.088 (7)	0.056 (7)	0.063 (6)	0.012 (5)	0.031 (5)	0.017 (4)
ON4B	0.111 (6)	0.091 (6)	0.073 (5)	0.003 (5)	0.051 (4)	0.023 (4)
ON5B	0.093 (10)	0.119 (13)	0.104 (10)	0.026 (9)	0.025 (8)	0.024 (8)
ON6B	0.116 (7)	0.116 (7)	0.060 (5)	−0.001 (6)	0.030 (4)	0.035 (5)
NC1	0.0494 (17)	0.0560 (18)	0.0535 (17)	0.0200 (14)	0.0160 (14)	0.0325 (15)
C11	0.057 (2)	0.055 (2)	0.066 (3)	0.0146 (18)	0.015 (2)	0.0149 (19)
C12	0.073 (3)	0.093 (3)	0.076 (3)	0.024 (3)	0.039 (3)	0.021 (3)
C13	0.057 (3)	0.110 (4)	0.133 (5)	0.014 (3)	0.049 (3)	0.037 (4)
C14	0.055 (3)	0.075 (3)	0.121 (4)	−0.004 (2)	0.022 (3)	0.019 (3)
C15	0.065 (3)	0.066 (3)	0.060 (3)	0.017 (2)	0.000 (2)	0.011 (2)
NC2	0.0444 (16)	0.0531 (17)	0.0442 (16)	0.0118 (13)	0.0191 (13)	0.0071 (13)
C21	0.051 (2)	0.047 (2)	0.073 (3)	0.0094 (17)	0.020 (2)	0.0117 (19)
C22	0.056 (2)	0.070 (3)	0.074 (3)	0.015 (2)	0.011 (2)	0.035 (2)
C23	0.045 (2)	0.065 (3)	0.082 (3)	0.0108 (19)	0.005 (2)	0.019 (2)
C24	0.043 (2)	0.053 (2)	0.085 (3)	0.0018 (17)	0.010 (2)	0.011 (2)
C25	0.063 (3)	0.066 (3)	0.084 (3)	0.013 (2)	0.041 (2)	0.026 (2)
NC3	0.0533 (19)	0.0462 (17)	0.0531 (18)	−0.0058 (14)	0.0070 (15)	−0.0004 (14)
C31	0.073 (3)	0.052 (2)	0.078 (3)	0.009 (2)	0.035 (2)	0.001 (2)
C32	0.080 (3)	0.066 (3)	0.093 (3)	0.027 (2)	0.037 (3)	0.036 (3)
C33	0.112 (4)	0.071 (3)	0.103 (4)	0.017 (3)	0.051 (3)	0.047 (3)
C34	0.105 (4)	0.086 (3)	0.083 (3)	0.023 (3)	0.056 (3)	0.030 (3)
C35	0.066 (3)	0.076 (3)	0.105 (4)	0.012 (2)	0.047 (3)	0.018 (3)
NC4	0.067 (2)	0.054 (2)	0.111 (3)	−0.0089 (18)	0.025 (2)	0.027 (2)
C41	0.063 (3)	0.074 (4)	0.149 (6)	−0.007 (3)	−0.009 (4)	0.028 (4)
C42	0.119 (5)	0.084 (4)	0.097 (4)	0.003 (4)	−0.013 (4)	0.032 (3)
C43	0.145 (6)	0.105 (5)	0.066 (3)	0.008 (4)	0.012 (4)	0.002 (3)
C44	0.095 (4)	0.091 (4)	0.088 (4)	0.022 (3)	0.020 (3)	0.012 (3)
C45	0.087 (4)	0.060 (3)	0.093 (4)	0.006 (3)	0.004 (3)	0.018 (3)
NC5	0.090 (8)	0.066 (8)	0.049 (7)	0.001 (6)	0.019 (6)	0.002 (8)
C51	0.069 (8)	0.056 (10)	0.072 (11)	0.005 (7)	0.024 (7)	0.002 (9)
C52	0.060 (7)	0.056 (9)	0.038 (11)	−0.016 (8)	0.014 (8)	0.009 (10)
C53	0.071 (7)	0.045 (7)	0.039 (6)	0.010 (5)	0.019 (5)	0.020 (6)
C54	0.051 (7)	0.083 (12)	0.048 (10)	0.000 (8)	0.015 (7)	0.017 (12)
C55	0.083 (8)	0.069 (9)	0.041 (9)	−0.022 (8)	0.020 (7)	0.010 (8)
NC6	0.084 (11)	0.044 (9)	0.061 (11)	0.015 (9)	0.037 (9)	0.012 (9)
C61	0.057 (10)	0.040 (13)	0.055 (16)	−0.019 (10)	−0.010 (12)	−0.015 (14)
C62	0.083 (11)	0.046 (12)	0.037 (13)	−0.008 (11)	0.033 (9)	0.003 (12)
C63	0.100 (14)	0.047 (11)	0.038 (11)	0.012 (10)	0.042 (9)	−0.005 (10)
C64	0.075 (11)	0.058 (14)	0.068 (17)	0.006 (12)	0.009 (12)	0.018 (13)
C65	0.061 (10)	0.059 (12)	0.041 (17)	−0.007 (12)	0.022 (12)	−0.006 (16)

### Geometric parameters (Å, °)

**Table:**

V1—O12	1.608 (2)	C23—C24	1.528 (6)
V1—O8^i^	1.795 (2)	C23—H23A	0.9700
V1—O5^i^	1.849 (2)	C23—H23B	0.9700
V1—O1	1.978 (2)	C24—C25	1.502 (6)
V1—O14	2.067 (2)	C24—H24A	0.9700
V1—O13	2.277 (2)	C24—H24B	0.9700
V1—V3	3.1180 (8)	C25—H25A	0.9700
V2—O2	1.687 (2)	C25—H25B	0.9700
V2—O3	1.692 (2)	NC3—C35	1.472 (5)
V2—O1	1.914 (2)	NC3—C31	1.482 (5)
V2—O14^i^	2.003 (2)	NC3—HC3A	0.9000
V2—O13	2.081 (2)	NC3—HC3B	0.9000
V2—O13^i^	2.1346 (19)	C31—C32	1.493 (6)
V2—V5^i^	3.0747 (8)	C31—H31A	0.9700
V2—V4	3.0887 (8)	C31—H31B	0.9700
V3—O11	1.606 (2)	C32—C33	1.515 (6)
V3—O4	1.793 (2)	C32—H32A	0.9700
V3—O7	1.861 (2)	C32—H32B	0.9700
V3—O1	1.981 (2)	C33—C34	1.519 (6)
V3—O14	2.059 (2)	C33—H33A	0.9700
V3—O13^i^	2.2672 (19)	C33—H33B	0.9700
V3—V5	3.1193 (8)	C34—C35	1.506 (7)
V4—O10	1.602 (2)	C34—H34A	0.9700
V4—O6	1.845 (2)	C34—H34B	0.9700
V4—O5	1.850 (2)	C35—H35A	0.9700
V4—O4	1.929 (2)	C35—H35B	0.9700
V4—O3	2.014 (2)	NC4—C45	1.468 (6)
V4—O13^i^	2.345 (2)	NC4—C41	1.502 (7)
V4—V5	3.0906 (9)	NC4—HC4A	0.9000
V5—O9	1.606 (2)	NC4—HC4B	0.9000
V5—O6	1.830 (2)	C41—C42	1.469 (8)
V5—O7	1.846 (2)	C41—H41A	0.9700
V5—O8	1.922 (2)	C41—H41B	0.9700
V5—O2^i^	2.059 (2)	C42—C43	1.521 (8)
V5—O13^i^	2.3366 (19)	C42—H42A	0.9700
V5—V2^i^	3.0747 (8)	C42—H42B	0.9700
O2—V5^i^	2.059 (2)	C43—C44	1.542 (7)
O5—V1^i^	1.849 (2)	C43—H43A	0.9700
O8—V1^i^	1.795 (2)	C43—H43B	0.9700
O13—V2^i^	2.1346 (19)	C44—C45	1.482 (7)
O13—V3^i^	2.2672 (19)	C44—H44A	0.9700
O13—V5^i^	2.3366 (19)	C44—H44B	0.9700
O13—V4^i^	2.345 (2)	C45—H45A	0.9700
O14—V2^i^	2.003 (2)	C45—H45B	0.9700
NO1—ON1	1.224 (5)	NC5—C51	1.475 (12)
NO1—ON2	1.229 (6)	NC5—C55	1.477 (11)
NO1—ON3	1.244 (6)	NC5—HC5A	0.9000
NO2A—ON6A	1.224 (8)	NC5—HC5B	0.9000
NO2A—ON5A	1.233 (8)	C51—C52	1.524 (14)
NO2A—ON4A	1.243 (8)	C51—H51A	0.9700
NO2B—ON6B	1.227 (8)	C51—H51B	0.9700
NO2B—ON4B	1.232 (8)	C52—C53	1.503 (12)
NO2B—ON5B	1.241 (8)	C52—H52A	0.9700
NC1—C11	1.484 (4)	C52—H52B	0.9700
NC1—C15	1.486 (5)	C53—C54	1.515 (12)
NC1—HC1A	0.9000	C53—H53A	0.9700
NC1—HC1B	0.9000	C53—H53B	0.9700
C11—C12	1.504 (6)	C54—C55	1.503 (15)
C11—H11A	0.9700	C54—H54A	0.9700
C11—H11B	0.9700	C54—H54B	0.9700
C12—C13	1.524 (6)	C55—H55A	0.9700
C12—H12A	0.9700	C55—H55B	0.9700
C12—H12B	0.9700	NC6—C61	1.474 (17)
C13—C14	1.521 (6)	NC6—C65	1.482 (16)
C13—H13A	0.9700	NC6—HC6A	0.9000
C13—H13B	0.9700	NC6—HC6B	0.9000
C14—C15	1.499 (6)	C61—C62	1.516 (17)
C14—H14A	0.9700	C61—H61A	0.9700
C14—H14B	0.9700	C61—H61B	0.9700
C15—H15A	0.9700	C62—C63	1.508 (17)
C15—H15B	0.9700	C62—H62A	0.9700
NC2—C25	1.485 (5)	C62—H62B	0.9700
NC2—C21	1.491 (5)	C63—C64	1.535 (17)
NC2—HC2A	0.9000	C63—H63A	0.9700
NC2—HC2B	0.9000	C63—H63B	0.9700
C21—C22	1.506 (6)	C64—C65	1.514 (17)
C21—H21A	0.9700	C64—H64A	0.9700
C21—H21B	0.9700	C64—H64B	0.9700
C22—C23	1.525 (6)	C65—H65A	0.9700
C22—H22A	0.9700	C65—H65B	0.9700
C22—H22B	0.9700		
			
O12—V1—O8^i^	104.18 (11)	C14—C13—H13A	109.7
O12—V1—O5^i^	101.89 (11)	C12—C13—H13A	109.7
O8^i^—V1—O5^i^	95.28 (10)	C14—C13—H13B	109.7
O12—V1—O1	100.96 (11)	C12—C13—H13B	109.7
O8^i^—V1—O1	92.68 (9)	H13A—C13—H13B	108.2
O5^i^—V1—O1	153.11 (9)	C15—C14—C13	112.1 (4)
O12—V1—O14	99.69 (10)	C15—C14—H14A	109.2
O8^i^—V1—O14	155.02 (9)	C13—C14—H14A	109.2
O5^i^—V1—O14	86.82 (9)	C15—C14—H14B	109.2
O1—V1—O14	75.51 (8)	C13—C14—H14B	109.2
O12—V1—O13	174.38 (10)	H14A—C14—H14B	107.9
O8^i^—V1—O13	80.74 (8)	NC1—C15—C14	109.4 (4)
O5^i^—V1—O13	80.13 (8)	NC1—C15—H15A	109.8
O1—V1—O13	75.87 (7)	C14—C15—H15A	109.8
O14—V1—O13	75.10 (7)	NC1—C15—H15B	109.8
O12—V1—V3	90.74 (9)	C14—C15—H15B	109.8
O8^i^—V1—V3	130.72 (7)	H15A—C15—H15B	108.3
O5^i^—V1—V3	127.63 (7)	C25—NC2—C21	112.5 (3)
O1—V1—V3	38.07 (6)	C25—NC2—HC2A	109.1
O14—V1—V3	40.83 (6)	C21—NC2—HC2A	109.1
O13—V1—V3	83.92 (5)	C25—NC2—HC2B	109.1
O2—V2—O3	107.57 (11)	C21—NC2—HC2B	109.1
O2—V2—O1	99.52 (9)	HC2A—NC2—HC2B	107.8
O3—V2—O1	97.77 (10)	NC2—C21—C22	109.9 (3)
O2—V2—O14^i^	95.78 (9)	NC2—C21—H21A	109.7
O3—V2—O14^i^	94.86 (10)	C22—C21—H21A	109.7
O1—V2—O14^i^	156.18 (9)	NC2—C21—H21B	109.7
O2—V2—O13	88.43 (9)	C22—C21—H21B	109.7
O3—V2—O13	163.72 (9)	H21A—C21—H21B	108.2
O1—V2—O13	82.11 (8)	C21—C22—C23	111.9 (4)
O14^i^—V2—O13	80.16 (8)	C21—C22—H22A	109.2
O2—V2—O13^i^	165.82 (9)	C23—C22—H22A	109.2
O3—V2—O13^i^	86.29 (9)	C21—C22—H22B	109.2
O1—V2—O13^i^	81.06 (8)	C23—C22—H22B	109.2
O14^i^—V2—O13^i^	79.69 (8)	H22A—C22—H22B	107.9
O13—V2—O13^i^	77.59 (8)	C22—C23—C24	109.4 (3)
O2—V2—V5^i^	39.01 (7)	C22—C23—H23A	109.8
O3—V2—V5^i^	146.50 (8)	C24—C23—H23A	109.8
O1—V2—V5^i^	92.17 (6)	C22—C23—H23B	109.8
O14^i^—V2—V5^i^	88.30 (6)	C24—C23—H23B	109.8
O13—V2—V5^i^	49.42 (5)	H23A—C23—H23B	108.2
O13^i^—V2—V5^i^	126.97 (6)	C25—C24—C23	111.8 (3)
O2—V2—V4	144.46 (8)	C25—C24—H24A	109.3
O3—V2—V4	36.99 (7)	C23—C24—H24A	109.3
O1—V2—V4	90.73 (6)	C25—C24—H24B	109.3
O14^i^—V2—V4	87.29 (6)	C23—C24—H24B	109.3
O13—V2—V4	126.83 (5)	H24A—C24—H24B	107.9
O13^i^—V2—V4	49.30 (5)	NC2—C25—C24	109.4 (3)
V5^i^—V2—V4	174.76 (2)	NC2—C25—H25A	109.8
O11—V3—O4	103.86 (11)	C24—C25—H25A	109.8
O11—V3—O7	101.51 (11)	NC2—C25—H25B	109.8
O4—V3—O7	95.26 (10)	C24—C25—H25B	109.8
O11—V3—O1	100.88 (10)	H25A—C25—H25B	108.2
O4—V3—O1	92.61 (9)	C35—NC3—C31	114.1 (4)
O7—V3—O1	153.70 (9)	C35—NC3—HC3A	108.7
O11—V3—O14	99.32 (11)	C31—NC3—HC3A	108.7
O4—V3—O14	155.65 (9)	C35—NC3—HC3B	108.7
O7—V3—O14	87.27 (9)	C31—NC3—HC3B	108.7
O1—V3—O14	75.62 (8)	HC3A—NC3—HC3B	107.6
O11—V3—O13^i^	173.84 (10)	NC3—C31—C32	110.0 (3)
O4—V3—O13^i^	81.88 (8)	NC3—C31—H31A	109.7
O7—V3—O13^i^	79.94 (8)	C32—C31—H31A	109.7
O1—V3—O13^i^	76.38 (7)	NC3—C31—H31B	109.7
O14—V3—O13^i^	74.71 (8)	C32—C31—H31B	109.7
O11—V3—V1	90.45 (9)	H31A—C31—H31B	108.2
O4—V3—V1	130.59 (7)	C31—C32—C33	111.6 (4)
O7—V3—V1	128.29 (7)	C31—C32—H32A	109.3
O1—V3—V1	37.99 (6)	C33—C32—H32A	109.3
O14—V3—V1	41.02 (6)	C31—C32—H32B	109.3
O13^i^—V3—V1	84.03 (5)	C33—C32—H32B	109.3
O11—V3—V5	133.84 (8)	H32A—C32—H32B	108.0
O4—V3—V5	83.23 (7)	C32—C33—C34	111.2 (4)
O7—V3—V5	32.56 (6)	C32—C33—H33A	109.4
O1—V3—V5	124.61 (6)	C34—C33—H33A	109.4
O14—V3—V5	86.12 (6)	C32—C33—H33B	109.4
O13^i^—V3—V5	48.29 (5)	C34—C33—H33B	109.4
V1—V3—V5	119.61 (2)	H33A—C33—H33B	108.0
O10—V4—O6	103.65 (12)	C35—C34—C33	112.0 (4)
O10—V4—O5	103.03 (12)	C35—C34—H34A	109.2
O6—V4—O5	92.59 (10)	C33—C34—H34A	109.2
O10—V4—O4	100.98 (12)	C35—C34—H34B	109.2
O6—V4—O4	88.62 (10)	C33—C34—H34B	109.2
O5—V4—O4	154.94 (9)	H34A—C34—H34B	107.9
O10—V4—O3	101.07 (11)	NC3—C35—C34	109.5 (4)
O6—V4—O3	154.99 (9)	NC3—C35—H35A	109.8
O5—V4—O3	85.48 (9)	C34—C35—H35A	109.8
O4—V4—O3	82.99 (9)	NC3—C35—H35B	109.8
O10—V4—O13^i^	174.85 (11)	C34—C35—H35B	109.8
O6—V4—O13^i^	81.17 (8)	H35A—C35—H35B	108.2
O5—V4—O13^i^	78.32 (8)	C45—NC4—C41	112.1 (4)
O4—V4—O13^i^	77.15 (8)	C45—NC4—HC4A	109.2
O3—V4—O13^i^	74.01 (8)	C41—NC4—HC4A	109.2
O10—V4—V2	131.42 (10)	C45—NC4—HC4B	109.2
O6—V4—V2	124.76 (7)	C41—NC4—HC4B	109.2
O5—V4—V2	80.44 (7)	HC4A—NC4—HC4B	107.9
O4—V4—V2	78.37 (6)	C42—C41—NC4	110.5 (4)
O3—V4—V2	30.36 (6)	C42—C41—H41A	109.5
O13^i^—V4—V2	43.65 (5)	NC4—C41—H41A	109.5
O10—V4—V5	136.23 (10)	C42—C41—H41B	109.5
O6—V4—V5	32.60 (7)	NC4—C41—H41B	109.5
O5—V4—V5	85.55 (7)	H41A—C41—H41B	108.1
O4—V4—V5	82.07 (7)	C41—C42—C43	112.1 (5)
O3—V4—V5	122.51 (6)	C41—C42—H42A	109.2
O13^i^—V4—V5	48.57 (5)	C43—C42—H42A	109.2
V2—V4—V5	92.184 (19)	C41—C42—H42B	109.2
O9—V5—O6	103.89 (12)	C43—C42—H42B	109.2
O9—V5—O7	102.81 (11)	H42A—C42—H42B	107.9
O6—V5—O7	93.08 (10)	C42—C43—C44	109.9 (5)
O9—V5—O8	101.31 (11)	C42—C43—H43A	109.7
O6—V5—O8	89.64 (10)	C44—C43—H43A	109.7
O7—V5—O8	154.30 (9)	C42—C43—H43B	109.7
O9—V5—O2^i^	100.73 (11)	C44—C43—H43B	109.7
O6—V5—O2^i^	155.14 (9)	H43A—C43—H43B	108.2
O7—V5—O2^i^	84.73 (9)	C45—C44—C43	111.4 (5)
O8—V5—O2^i^	82.19 (9)	C45—C44—H44A	109.3
O9—V5—O13^i^	174.15 (11)	C43—C44—H44A	109.3
O6—V5—O13^i^	81.70 (8)	C45—C44—H44B	109.3
O7—V5—O13^i^	78.39 (8)	C43—C44—H44B	109.3
O8—V5—O13^i^	76.73 (8)	H44A—C44—H44B	108.0
O2^i^—V5—O13^i^	73.60 (7)	NC4—C45—C44	111.7 (4)
O9—V5—V2^i^	131.76 (10)	NC4—C45—H45A	109.3
O6—V5—V2^i^	124.20 (7)	C44—C45—H45A	109.3
O7—V5—V2^i^	80.10 (6)	NC4—C45—H45B	109.3
O8—V5—V2^i^	77.28 (6)	C44—C45—H45B	109.3
O2^i^—V5—V2^i^	31.04 (6)	H45A—C45—H45B	108.0
O13^i^—V5—V2^i^	42.56 (5)	C51—NC5—C55	112.7 (11)
O9—V5—V4	136.76 (10)	C51—NC5—HC5A	109.0
O6—V5—V4	32.90 (7)	C55—NC5—HC5A	109.0
O7—V5—V4	86.10 (7)	C51—NC5—HC5B	109.0
O8—V5—V4	82.40 (7)	C55—NC5—HC5B	109.0
O2^i^—V5—V4	122.33 (6)	HC5A—NC5—HC5B	107.8
O13^i^—V5—V4	48.80 (5)	NC5—C51—C52	109.3 (11)
V2^i^—V5—V4	91.317 (19)	NC5—C51—H51A	109.8
O9—V5—V3	135.45 (9)	C52—C51—H51A	109.8
O6—V5—V3	81.46 (7)	NC5—C51—H51B	109.8
O7—V5—V3	32.87 (6)	C52—C51—H51B	109.8
O8—V5—V3	123.11 (6)	H51A—C51—H51B	108.3
O2^i^—V5—V3	83.46 (6)	C53—C52—C51	105.9 (10)
O13^i^—V5—V3	46.42 (5)	C53—C52—H52A	110.6
V2^i^—V5—V3	63.252 (17)	C51—C52—H52A	110.6
V4—V5—V3	60.646 (19)	C53—C52—H52B	110.6
V2—O1—V1	106.75 (9)	C51—C52—H52B	110.6
V2—O1—V3	107.80 (9)	H52A—C52—H52B	108.7
V1—O1—V3	103.94 (10)	C52—C53—C54	113.5 (11)
V2—O2—V5^i^	109.95 (11)	C52—C53—H53A	108.9
V2—O3—V4	112.65 (11)	C54—C53—H53A	108.9
V3—O4—V4	114.73 (10)	C52—C53—H53B	108.9
V1^i^—O5—V4	115.14 (11)	C54—C53—H53B	108.9
V5—O6—V4	114.50 (11)	H53A—C53—H53B	107.7
V5—O7—V3	114.57 (11)	C55—C54—C53	107.3 (10)
V1^i^—O8—V5	115.62 (10)	C55—C54—H54A	110.3
V2—O13—V2^i^	102.41 (8)	C53—C54—H54A	110.3
V2—O13—V3^i^	96.58 (8)	C55—C54—H54B	110.3
V2^i^—O13—V3^i^	91.23 (7)	C53—C54—H54B	110.3
V2—O13—V1	91.44 (7)	H54A—C54—H54B	108.5
V2^i^—O13—V1	95.85 (8)	NC5—C55—C54	108.9 (11)
V3^i^—O13—V1	167.94 (9)	NC5—C55—H55A	109.9
V2—O13—V5^i^	88.02 (7)	C54—C55—H55A	109.9
V2^i^—O13—V5^i^	169.33 (10)	NC5—C55—H55B	109.9
V3^i^—O13—V5^i^	85.29 (7)	C54—C55—H55B	109.9
V1—O13—V5^i^	86.00 (6)	H55A—C55—H55B	108.3
V2—O13—V4^i^	170.19 (9)	C61—NC6—C65	112.0 (19)
V2^i^—O13—V4^i^	87.06 (7)	C61—NC6—HC6A	109.2
V3^i^—O13—V4^i^	85.64 (6)	C65—NC6—HC6A	109.2
V1—O13—V4^i^	84.98 (7)	C61—NC6—HC6B	109.2
V5^i^—O13—V4^i^	82.63 (6)	C65—NC6—HC6B	109.2
V2^i^—O14—V3	106.17 (9)	HC6A—NC6—HC6B	107.9
V2^i^—O14—V1	107.18 (9)	NC6—C61—C62	107.0 (18)
V3—O14—V1	98.15 (9)	NC6—C61—H61A	110.3
ON1—NO1—ON2	122.1 (7)	C62—C61—H61A	110.3
ON1—NO1—ON3	119.3 (6)	NC6—C61—H61B	110.3
ON2—NO1—ON3	118.7 (5)	C62—C61—H61B	110.3
ON6A—NO2A—ON5A	120.8 (8)	H61A—C61—H61B	108.6
ON6A—NO2A—ON4A	119.7 (7)	C63—C62—C61	109.3 (18)
ON5A—NO2A—ON4A	119.5 (7)	C63—C62—H62A	109.8
ON6B—NO2B—ON4B	120.1 (7)	C61—C62—H62A	109.8
ON6B—NO2B—ON5B	119.9 (8)	C63—C62—H62B	109.8
ON4B—NO2B—ON5B	120.0 (8)	C61—C62—H62B	109.8
C11—NC1—C15	113.1 (3)	H62A—C62—H62B	108.3
C11—NC1—HC1A	109.0	C62—C63—C64	107.7 (17)
C15—NC1—HC1A	109.0	C62—C63—H63A	110.2
C11—NC1—HC1B	109.0	C64—C63—H63A	110.2
C15—NC1—HC1B	109.0	C62—C63—H63B	110.2
HC1A—NC1—HC1B	107.8	C64—C63—H63B	110.2
NC1—C11—C12	110.0 (3)	H63A—C63—H63B	108.5
NC1—C11—H11A	109.7	C65—C64—C63	107.7 (18)
C12—C11—H11A	109.7	C65—C64—H64A	110.2
NC1—C11—H11B	109.7	C63—C64—H64A	110.2
C12—C11—H11B	109.7	C65—C64—H64B	110.2
H11A—C11—H11B	108.2	C63—C64—H64B	110.2
C11—C12—C13	110.9 (4)	H64A—C64—H64B	108.5
C11—C12—H12A	109.5	NC6—C65—C64	108.5 (18)
C13—C12—H12A	109.5	NC6—C65—H65A	110.0
C11—C12—H12B	109.5	C64—C65—H65A	110.0
C13—C12—H12B	109.5	NC6—C65—H65B	110.0
H12A—C12—H12B	108.0	C64—C65—H65B	110.0
C14—C13—C12	109.9 (4)	H65A—C65—H65B	108.4

Symmetry codes: (i) −*x*+1, −*y*+1, −*z*+1.

### Hydrogen-bond geometry (Å, °)

**Table:**

*D*—H···*A*	*D*—H	H···*A*	*D*···*A*	*D*—H···*A*
NC1—HC1A···O8	0.90	1.80	2.693 (3)	174
NC1—HC1B···ON6A^ii^	0.90	1.95	2.829 (8)	164
NC1—HC1B···ON6B	0.90	2.05	2.876 (9)	153
NC1—HC1B···ON5A^ii^	0.90	2.48	3.214 (12)	139
NC1—HC1B···ON5B	0.90	2.54	3.355 (13)	151
NC2—HC2A···ON4A	0.90	1.95	2.824 (7)	164
NC2—HC2A···ON4B^ii^	0.90	2.05	2.909 (8)	159
NC2—HC2A···ON5B^ii^	0.90	2.50	3.280 (13)	145
NC2—HC2A···ON5A	0.90	2.56	3.251 (12)	134
NC2—HC2B···O4	0.90	1.85	2.746 (3)	174
NC3—HC3A···O5^i^	0.90	1.85	2.749 (4)	175
NC3—HC3B···ON3	0.90	2.06	2.885 (5)	152
NC3—HC3B···ON2	0.90	2.31	3.095 (5)	145
NC4—HC4A···O7	0.90	1.82	2.716 (4)	172
NC4—HC4B···ON3	0.90	2.16	2.954 (6)	147
NC4—HC4B···ON1	0.90	2.26	3.060 (6)	148
NC5—HC5A···O6	0.90	2.40	3.248 (17)	158
NC6—HC6A···ON4B	0.90	2.12	2.92 (2)	147
NC6—HC6B···O6^ii^	0.90	1.92	2.80 (2)	166

Symmetry codes: (ii) −*x*+1, −*y*+1, −*z*; (i) −*x*+1, −*y*+1, −*z*+1.
